# A Distance-Based Neurorehabilitation Evaluation Method Using Linear SVM and Resting-State fMRI

**DOI:** 10.3389/fneur.2019.01105

**Published:** 2019-11-01

**Authors:** Yunxiang Ge, Yu Pan, Qiong Wu, Weibei Dou

**Affiliations:** ^1^Department of Electronic Engineering, Tsinghua University, Beijing, China; ^2^Beijing National Research Center for Information Science and Technology (BNRist), Beijing, China; ^3^School of Clinical Medicine, Tsinghua University, Beijing, China; ^4^Department of Rehabilitation, Beijing Tsinghua Changgung Hospital, Beijing, China

**Keywords:** resting-state fMRI, functional connectivity, neurorehabilitation, support vector machine, spinal cord injury

## Abstract

During neurorehabilitation, clinical measurements are widely adopted to evaluate behavioral improvements after treatment. However, it is not able to identify or monitor the change of central nervous system (CNS) of each individual patient. Resting-state functional magnetic resonance imaging (rs-fMRI) has been widely used to investigate brain functions in healthy controls (HCs) and patients with neurological diseases, which could find functional changes following neurorehabilitation. In this paper, a distance-based rehabilitation evaluation method based on rs-fMRI was proposed. Specifically, we posit that in the functional connectivity (FC) space, patients and HCs distribute separately. Linear support vector machines (SVM) were trained on the brain networks to firstly separate patients from HCs. Second, the FC similarity between patients and HCs was measured by the L2 distance of each subject's feature vector to the separating hyperplane. Finally, statistical analysis of the distance revealed rehabilitation program induced improvements in patients and predicted rehabilitation outcomes. An rs-fMRI dataset with 22 HCs and 18 spinal cord injury (SCI) patients was utilized to validate our method. We built whole-brain networks using five atlases to test the robustness of the method and search for features under different node resolutions. The classifier successfully separated patients and HCs. Significant improvements in FC after treatment were found for the patients for all five atlases using the proposed method, which was consistent with clinical measurements. Furthermore, distance obtained from individual patient's longitudinal data showed a similar trend with each one's clinical scores, implying the possibility of individual rehabilitation outcome tracking and prediction. Our method not only provides a novel perspective of applying rs-fMRI to neurorehabilitation monitoring but also proves the potential in individualized rehabilitation prediction.

## Introduction

Neurorehabilitation aims to help patients with central nervous system (CNS) disease regain certain lost abilities and finally return to home and society. Spinal cord injury (SCI) is a common CNS illness that highly influences patients' daily life and brings heavy burden to the patient's family (1–6). Typically, a rehabilitation program lasts for weeks or months. One crucial problem is how to monitor the rehabilitation progress of each patient. Currently, most clinics use clinical measurements such as the International Standards for the Neurological Classification of Spinal Cord Injury (7, 8), Fugl–Meyer Assessment, Wolf Motor Function Test, and Action Research Arm Test (5, 9) to evaluate how patients perform in several function-related tasks. The score rating, however, reflects the behavioral improvements of patients. We wonder if there is another way to monitor the changes in CNS induced by treatment, specifically functional changes in the brain. Since clinical scores can be viewed as a distance measuring how similar patients perform as healthy subjects, is it possible to calculate a distance between patients and healthy subjects from neuroimaging data?

Resting-state functional magnetic resonance imaging (rs-fMRI) is a potential tool for clinical applications (10). Functional connectivity (FC), defined as temporal correlations between spatially distinct brain regions (11), has been used to analyze blood-oxygen level-dependent functional magnetic resonance imaging (BOLD fMRI). Based on fMRI of healthy subjects, several resting-state networks (RSNs), and intrinsic connectivity networks (ICNs) have been identified (12–16).

Apart from researches on healthy brain functions, rs-fMRI has also been applied to study diseases related to functional changes, including Alzheimer's disease, dementia, schizophrenia, and depression [see (10) for a review]. Researchers found diseases related to FC changes and brain abnormalities (17–19), which may lead to the discovery of disease-specific biomarkers. Zeng et al. (20) applied a multivariate pattern analysis on the whole-brain resting-state FC patterns to identify major depressive individuals from healthy controls (HCs). Previously, brain connectivity changes in SCI patients were evaluated focusing on the sensorimotor network using rs-fMRI (21–24). FC analyses were also performed using network-based statistic (NBS) (25), RSNs (26), and graph theory (27), as well as combining with structural reorganizations (28). However, from these findings of alterations in functional features, we can hardly tell whether patients became healthier after treatment or predict how well patients would recover.

In order to answer the above questions, we propose a distance-based rehabilitation evaluation method. The distance is calculated in a classification framework. One of the most prevailing classifiers is the support vector machine (SVM). It tries to find an optimal separating hyperplane that achieves maximal margin between the two classes (29). Using fMRI data, studies have demonstrated that SVM can successfully classify cognitive states (30–32) and patients with depression (33, 34). In these studies, features used to train the SVM include FC and graph theory properties. Apart from non-linear kernel SVM, the original linear SVM works well when using correlation coefficients as features (33). Moreover, the SVM provides an ideal framework to quantify the difference between samples of the two classes, since the geometry interpretation of linear SVM is simple and clear. However, the performance of linear SVM on SCI patients is not fully investigated before.

Here, we used multi-session resting-state fMRI data of 18 SCI patients and 22 HCs to validate our method. The rest of this article is organized as follows: we elaborate our method in the second section, describe the materials and data processing details in the third section, and present the experiment results in the fourth section, followed by discussion and conclusion in fifth and sixth sections.

## Method

**Figure 1:**
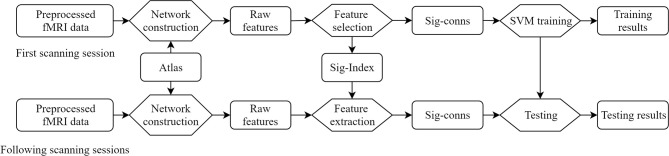
Overview of the framework. Sig-Index, significant index; Sig-conns, significant connections.

The proposed method is summarized in Figure 1. Concretely, the raw features, defined as FC between brain regions, are obtained by building brain networks with brain atlas on the first scanning sessions. A feature selection procedure eliminates insignificant features and generates a set of indices of significant features. Each subject is represented by a vector of these significant features in the feature space. Linear SVMs were trained to separate patients and HCs. The longitudinal fMRI data go through the same network construction procedure, and features identified as significant are taken out, forming the vector representation of this session.

In order to evaluate treatment-related functional changes, we posit that the feature vectors of HCs and patients are distributed separately in the feature space. If patients were getting better, they would become more and more similar to healthy subjects, and the feature vector representing patients would move toward HCs. Thus, it is possible to measure how well the patient has become using the distance of feature vectors. If this distance had a consistent trend, it would imply that the patient had recovered after treatment.

### Network Construction

The FC network, *N*, is built for each scan of each subjects using brain atlas. After preprocessing, averaged time course is extracted in each region and pairwise Pearson correlation coefficients, *n*_*ij*_, are calculated as the FC between two regions. Since the method applies to tracking longitudinal changes of patients, the parcellation of connectivity networks should not vary across multiple sessions. Regions defined by atlas are stable compared to data-driven approaches. In addition, different atlases could provide a range of node definition resolution, as well as covering whole brain or local regions of interests according to the patient disease.

### SVM Training

To select features for classification, we utilized a *t*-test filter on the brain networks of patients and HCs. Formally, let *C* = {*c*_1_, *c*_2_, …, *c*_*n*_} denote the set of HCs. Let *P* = {*p*_1_, *p*_2_, …, *p*_*m*_} denote the set of patients. A brain network is denoted by *N*_*s*, 1_ = {*n*_*ij*_}_*k* × *k*_, where *s* ∈ *C* or *s* ∈ *P* denotes the subject and the latter one means the first scanning session. Patients might have multiple scanning sessions, whereas HCs only have one. We chose the first sessions, {*N*_*p*_1_, 1_, *N*_*p*_2_, 1_, …, *N*_*p*_*m*_, 1_} and {*N*_*c*_1_, 1_, *N*_*c*_2_, 1_, …, *N*_*c*_*n*_, 1_}, to train the SVM. The correlation coefficient *n*_*ij*_ was extracted from each network of HCs and patients, forming two groups. Two-tailed two-sample *t*-test (*P* < 0.05, uncorrected) was performed on the two groups to decide whether this connection, *n*_*ij*_, is significant. This procedure was performed for every connection in the network. All significant connections were extracted as features and the index tuple (*i, j*) is put into the significant set *Sig*_*set* = {…, (*i, j*), …}. As a result, each subject was represented as a feature vector *v* containing significant connections of this subject.

Linear SVM classifiers were trained on the selected significant connections to classify the patient group (labeled as +1) and the healthy group (labeled as −1). Linear SVM tries to find an optimal separating hyperplane that achieves the largest margin of separation (29). The problem is defined as

$$\begin{array}{l} \underset{w}{\min}\frac{1}{2}\|w{\|}^{2}+C\underset{i}{\sum }\xi_{i} \end{array}$$

Subject to $y_{i}\left(w^{T}v_{i}+b\right)\geq 1-\xi_{i}$ and ξ_*i*_ ≥ 0

where *w* is the weight vector, ξ_*i*_ is the slack variable for sample *i*, *y*_*i*_ is the label of sample *i* (1 for patients, −1 for controls), *v*_*i*_ is the feature vector, and *b* is a bias constant.

### Rehabilitation Evaluation

The SVM classifier trained on the first scanning session was used on the follow-up data. The same features are extracted from longitudinal sessions for patients, forming feature vectors. For each patient, the feature vector $v={\left[\ldots ,\;n_{ij},\;\ldots \right]}^{T}$ where (*i, j*) ∈ *Sig*_*set*. We calculated the distance of feature vectors to the separating hyperplane as a measurement of outcome evaluation. Formally, the separating hyperplane of linear SVM is determined by

$$\begin{array}{l} h\left(v\right)=w^{T}v+b \end{array}$$

Note that the predicted label is *y* = *sgn*(*w*^*T*^*v* + *b*). The distance of a feature vector to the separating hyperplane can be calculated as

$$\begin{array}{l} d=\frac{h\left(v\right)}{\left\|w\right\|} \end{array}$$

Since patients are positive samples (labeled as +1), the above formula gives a “signed distance.” Positive distance value indicates that the sample point lies on the same side with patients and vice versa. As a result, if the distance decreases, the sample point would move toward HCs, indicating improvements of the subject.

## Experiment

### Subjects

Forty subjects were engaged in this study, including 18 incomplete SCI patients and 22 HCs. The healthy subjects had no history of neurological disorder. Patients presenting with all of the following criteria were considered for study inclusion: age 18–70, normal cognitive function, without brain lesions or implantable devices, incomplete injury [C1-T12, ASIA C, or D (7)], subacute SCI and chronic SCI (time since injury >1 month and <12 months), and neurologic level above T_12_. Patients with one or more of the following conditions were excluded from this study: SCI relapse due to any reason, mental illness, seizures, and having other severe cardiovascular or neurologic disease (Table 1). All subjects provided their written informed consent to participate according to the Declaration of Helsinki. The study protocol was approved by the Ethics Committee of Beijing Tsinghua Changgung Hospital of China (IRB No. 2015-002).

**Table 1:** Subject demographics.

	**SCI patients (*n* = 18) Mean ± SD**	**Healthy controls (*n* = 22) Mean ± SD**	**Statistic value**	***P*-value**
Gender(male/female)	7/11	8/14	χ^2^ = 0.027	0.870
Age (years)	42.33 ± 16.65	37.18 ± 12.24	*t* = 1.097	0.279
Illness duration (months)	2.39 ± 1.53	–	–	–
AISA level	C(4), D(14)	–	–	–
Mean motion (first session, mm)	0.058± 0.038	0.038 ±0.033	*t* = 1.699	0.098
Mean motion (second session, mm)	0.050 ± 0.032	–	*t* = 1.006	0.330^*^
Lower limb movement (before)	32.56 ± 8.36	–	*t* = 6.253	<0.001^*^
Lower limb movement (after)	37.06 ± 7.74	-	Wilcoxon stat = 0	<0.001^*^
Sensory (before)	161.44 ± 32.45	–	*t* = 1.982	0.064^*^
Sensory (after)	163.00 ± 32.81	–	Wilcoxon stat = 0	0.042^*^
SCIM (before)	52.72 ± 15.77	–	*t* = 3.884	0.001^*^
SCIM (after)	59.00 ± 16.71	–	Wilcoxon stat = 0	<0.001^*^

*For clinical measurements, lower limb movement and sensory and SCIM scores before and after treatment were shown. Significant results were shown in bold (P < 0.05)*.

* *Paired tests were performed on the first (before) and second (after) session (treatment) data*.

The SCI patients received standard care for SCI rehabilitation at our institution. The rehabilitation protocol comprises 5 h of therapy a day, 5 days per week, lasting for 2 weeks. The therapy includes training in physical therapy for the lower extremities for SCI patients, activity of daily living, and fitness training. The rehabilitation outcomes, including motor function, sensory function, and daily life ability, were evaluated by American Spinal Injury Association (ASIA) criteria (7) and the Spinal Cord Independence Measure (35). Clinical measurements used in this study include lower limb movement score, sensory score, and SCIM (Table 1).

### Scanning

All SCI patients went through two MRI sessions, with an interval of 2 weeks. In order to investigate the longitudinal rehabilitation outcome, five patients received two more scanning (four sessions in total). Healthy subjects were scanned only once. The time of scanning (number of weeks passed) since the first session is shown in Table 2.

**Table 2:** Longitudinal scanning time since inclusion.

**Subjects**	**1st session (weeks)**	**2nd session (weeks)**	**3rd session (weeks)**	**4th session (weeks)**
HCs	0	–	–	–
SCI 1-13	0	2	–	–
SCI 14	0	2	6	25
SCI 15	0	2	6	14
SCI 16	0	2	4	6
SCI 17	0	2	15	17
SCI 18	0	2	7	49

*Numbers are weeks passed since the first scanning session. Healthy controls were only scanned once. SCI subjects 1–13 have two sessions. Subjects 14–18 have four sessions. HC, healthy control; SCI, spinal cord injury*.

The MRI scanning was performed at the Department of Radiology, Beijing Tsinghua Changgung Hospital. A GE 3.0T MR scanner (DISCOVERY MR750 model; General Electric American, Waukesha, WI, USA) was used to acquire MRI data. Participants were positioned supine and scanned using a standard 32-channel head-coil. fMRI data of resting-state blood oxygen level-dependent images are scanned using “Ax-BOLD-rest” series with a gradient echo-planar imaging (EPI) sequence [repetition time [TR] = 2000 ms, echo time [TE] = 30 ms, flip angle [FA] = 90°, pixel space = 3.5 mm^2^, slice thickness = 3.5 mm, spacing between slices = 4 mm, acquisition matrix = [64, 0, 0, 64], equivalent to in-plane resolution = 64 × 64, reconstruction diameter = 224 mm, 34 axial slices, and 240 temporal positions]. T1-weighted images (T1) are scanned using “Sag 3D T1BRAVO” series [TR = 8.21 ms, TE = 3.18 ms, FA = 8, voxel space = 1 mm^3^, spacing between slices = 1 mm, acquisition matrix = [0, 256, 256, 0], equivalent to 256 axial slices and 256 coronal slices]. The sagittal slice number depended on the head size of each subject, ranging from 156 to 174 mm, corresponding to the 36 subjects in this study. The reconstruction diameter was 256 mm. In this study, only the fMRI data were used.

### Processing

We used DPARSFA (36) toolbox to preprocess fMRI data. The first 10 time points were excluded to account for magnetization saturation effect and let the subjects be familiar with the environment. Head motion was corrected before normalizing the image to a 2-mm-isotropic BOLD EPI template in the Montreal Neurological Institute (MNI) 152 standard space. Motion parameters were inspected and compared to investigate for any inter-group differences. Similar to the criteria in (20) and (37), no subject exhibited excessive head motion during scan acquisition (>2.5 mm translation and/or > 2 rotation). Following the method used in (38), the mean motion between patient and HC group showed no significant difference (Table 1). The image was resampled to 3-mm isotropic voxels and spatially smoothed by a Gaussian kernel with 6-mm full width at half maximum (FWHM), followed by the removal of the linear trend and nuisance covariates, including head motions, cerebral fluid, and the global signal. Finally, the time course was filtered to keep signals in 0.01–0.08 Hz.

The data analysis procedure, including network construction, feature selection and SVM training and testing, was carried out using an in-house python-based software. We adopted five brain atlases to define nodes of networks. Firstly, the Brodmann atlas (39) with 82 brain regions was used. Since the original Brodmann atlas does not contain mapping for the cerebellum, which is related to functional changes after SCI (25), we also combined the cerebellum in the Automated Anatomical Labeling (AAL) atlas (40) (26 areas) into the Brodmann atlas, creating a 108-area parcellation (Brodmann_ce). The original AAL atlas was also used. Two recently proposed atlases were included as well. The AICHA atlas contains 384 brain regions in both hemispheres that highlight homotopy and maximal intrinsic connectivity between regions (41). The Brainnetome atlas was built using rs-fMRI and diffusion MRI of the Human Connectome Project, including 246 regions (42). All experiments were repeated for the five atlases and results were analyzed separately.

During SVM training, the parameter *C* controls the trade-off of misclassification and accuracy. We adjusted *C* and repeated the SVM training to investigate its influence on our method. The value of *C* was selected from [0.1, 0.5, 1, 5, 10, 50, 100, 500, 1000, 5000, 10,000].

**Figure 2:**
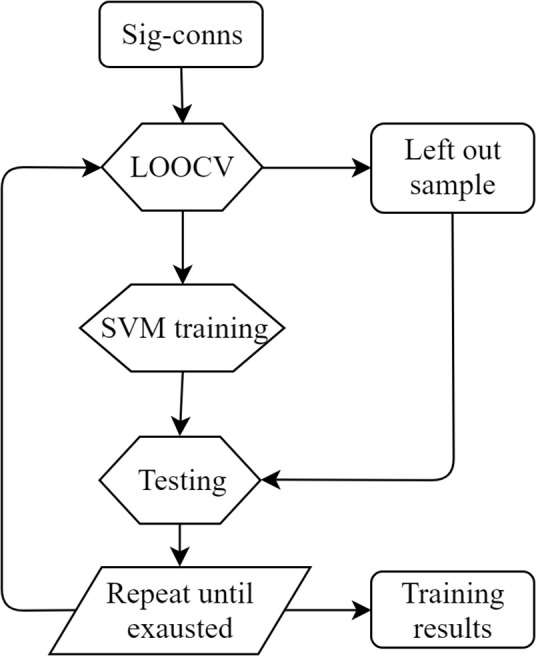
LOOCV training diagram. Sig-conns, significant connections; LOOCV, leave-one-out cross-validation.

Leave-one-out cross-validation (LOOCV) was applied to estimate the training accuracy (Figure 2). In the training process, one sample was left out as the test sample and the remaining data were used for training. The trained classifier was then used to classify the left-out sample. The accuracy was estimated by calculating the ratio of correctly classified samples against total sample amount. All SCI patients were used to test SVM performances. Precision, sensitivity, and specificity were also calculated. The definitions were as follows.

$$\begin{array}{lll} Precision & = & \frac{TP}{TP+FP}\\ Sensitivity & = & \frac{TP}{TP+FN}\\ Specificity & = & \frac{TN}{TN+FP} \end{array}$$

where TP stands for true positive (number of patients correctly classified as patient), FP stands for false positive (number of HCs incorrectly classified as patient), FN stands for false negative (number of patients incorrectly classified as HCs), and TN stands for true negative (number of HCs correctly classified as HCs). In order to test the significance of LOOCV accuracy, a permutation test was performed. The sample labels were randomly permuted 1,000 times to obtain an empirical *P*-value for the LOOCV accuracy.

For distance calculation in rehabilitation evaluation, we adopted the same LOOCV framework as described in the SVM training section. Specifically, during SVM training, one subject was selected as the evaluation subject. SVM was trained using the remaining subjects and the separating hyperplane was identified. Then, the distance of all sessions of the left-out evaluation subject was calculated as described in the Method section. This procedure was repeated for all subjects in order to obtain distance for each session of each subject. Besides, the five subjects with four MRI sessions were used to validate the proposed method in longitudinal recovery prediction. The distance of all four sessions was calculated and compared with clinical measurements. In order to compare distance and clinical scores, the two values were normalized to zero mean and unit variance according to the following formula.

$$\begin{array}{lll} d^{*}\left(i\right) & = & -1\times \frac{d\left(i\right)-mean\left(d\right)}{std\left(d\right)},\;i=1,\;2,\;3,\;4\\ s^{*}\left(i\right) & = & \frac{s\left(i\right)-mean\left(s\right)}{std\left(s\right)},\;i=1,\;2,\;3,\;4 \end{array}$$

where d* is normalized distance, s* is normalized scores, *mean*(·) stands for averaging, *std*(·) stands for standard deviation, and *i* represents session.

### Statistical Analysis

We performed intra-group analysis on distance and clinical measurements for each atlas and each SVM training parameter *C* separately. The statistical analysis was implemented using the SciPy python package. Specifically, paired *t*-test was performed on distance and clinical scores between the first and the second session scanning in the patient group to check for rehabilitation program induced improvements. Since the sample size is limited, we performed Shapiro Normality test on distance values and scores of both the first and the second session. Besides, we repeated the above-described analysis using non-parametric Wilcoxon signed-rank tests.

## Results

### SVM Training and Testing

**Figure A1:**
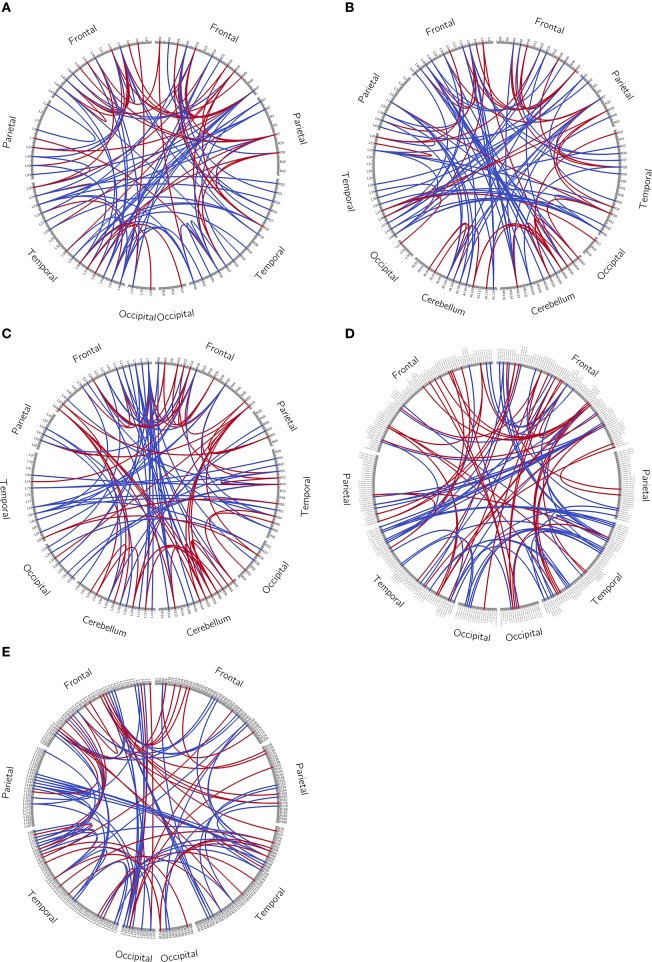
Circos plot. The whole brain was separated into frontal, parietal, temporal and occipital lobe, with or without cerebellum. Each brain region was arranged so that frontal regions appeared on the top of the graph. Only the most different 100 connections between the healthy control group and patient group (connections with highest absolute *t*-values during *t*-test) were shown. **(A)** Brodmann atlas; **(B)** Brodmann_ce atlas; **(C)** AAL atlas; **(D)** AICHA atlas; **(E)** Brainnetome atlas.

We trained an SVM classifier for each of the five atlases and each selection of the parameter *C*, using *t*-test filters and the LOOCV strategy. From our experiment, the training results were similar for all selections of *C*, and the mean LOOCV results for *C* = 1 are summarized in Table 3. All later reported results were based on this parameter setting. After *t*-test filtering, the percentage of significant connections for the SCI group was around 0.07. For illustration purposes, we plotted the significant connections using Circos^1^ (43). The graphs are shown in the Appendix (Figure A1).

**Table 3:** Training results.

	**Atlas**	**Discover**	**Accuracy**	***P* value**	**Precision**	**Sensitivity**	**Specificity**	**2nd test**
Feature selection	Brod.	0.0711	0.900	**<0.001**	0.8889	0.8889	0.9091	0.6667
	Brod.ce	0.0739	0.900	**<0.001**	0.8889	0.8889	0.9091	0.5556
	AAL	0.0699	0.925	**<0.001**	0.9412	0.8889	0.9545	0.7222
	AICHA	0.0669	1.000	**<0.001**	1.000	1.000	1.000	0.8333
	BN	0.0765	1.000	**<0.001**	1.000	1.000	1.000	0.8333
Whole brain	Brod.	–	0.5250	0.3816	0.4667	0.3889	0.6364	–
	Brod.ce	–	0.5000	0.4645	0.4375	0.3889	0.5909	–
	AAL	–	0.4750	0.5455	0.4211	0.4444	0.5000	–
	AICHA	–	0.6000	0.1269	0.5833	0.3889	0.7727	–
	BN	–	0.5750	0.1888	0.5385	0.3889	0.7273	–

*The training results were divided into with or without feature selection. The Discover column shows the ratio of significant connections to all possible connections. The last column shows the test accuracy when using the second session data as test data. This testing was only performed for the training with feature selection. Brod, Brodmann atlas; Brod.ce, Brodmann atlas with cerebellum; BN, Brainnetome atlas. Significant results were shown in bold*.

The LOOCV accuracies were above 0.9 for all atlases and empirically significant when tested with a 1,000 times permutation test (*P* < 0.001). Besides accuracy, the precision, sensitivity, and specificity were also reported in the table. The second session data were fed into the classifier as testing samples. The accuracy, which is the same as sensitivity, was reported in the last column in Table 3. In order to evaluate the influence of feature selection, we also tried to train the classifier without feature selection. That is, all whole-brain connections were used as features. Results for the whole-brain experiments are also presented in Table 3. The whole-brain results degraded drastically, with a non-significant (*P* > 0.05) accuracy around 0.5, which is similar to random guesses. This implies that the classification algorithm fails to classify samples without feature selection. Compared with the whole-brain result, the classifier can still segregate the second session data with relatively good accuracy (last column in Table 3).

We also used k-means algorithm on the selected significant connections to cluster samples without labels. The number of clusters was set to two and starting centroids were initialized randomly for 50 times in order to avoid local maxima (Table 4). The clustering accuracy was around 0.9 except for the Brodmann atlas, which achieved a 0.75 accuracy. We also reported the number of misclassified samples in Table 4. This result indicates that the selected feature vectors are indeed distributed differently in the feature space.

**Table 4:** Clustering results.

**Atlas**	**Brod**.	**Brod.ce**	**AAL**	**AICHA**	**BN**
Accuracy	0.75	0.89	0.94	0.94	0.86
Num. error	9	4	2	2	5

*K-means clustering results. Num. error represents the number of misclassified samples. Abbreviations are the same as in Table 3*.

### Distance Changes During Rehabilitation

We calculated distance for all scans of the patient group and performed intra-group analysis on distance and clinical scores. The Shapiro test was used to check the normality of distance value distribution of the first and the second session. Most tests showed no significant results. This indicated that distance values are mostly normally distributed. The paired *t*-tests and Wilcoxon signed-rank test results for intra-group tests (first minus second), based on SVM parameter *C* = 1, are shown in Table 5. Results of the parametric and non-parametric tests were the same. The distance of patient group was significantly decreased for all atlases (*P* < 0.05).

**Table 5:** Intra-group distance *t*-test results.

**Distance/Atlas**	**Brod**.	**Brod.ce**	**AAL**	**AICHA**	**BN**
HC	−0.527 ± 0.424	−0.855 ± 0.597	−1.057 ± 0.708	−3.524 ± 1.308	−2.222 ± 1.145
Patients before	0.565 ± 0.473	0.820 ± 0.630	0.843 ± 0.605	3.540 ± 1.899	2.044 ± 1.273
Patients after	0.123 ± 0.426	0.085 ± 0.577	0.126 ± 0.604	0.877 ± 1.015	0.462 ± 0.773
*t*-test *t*	**3.461**	**4.656**	**4.397**	**5.488**	**4.518**
*t*-test *p*	**0.003**	**<0.001**	**<0.001**	**<0.001**	**<0.001**
Wilcoxon stat	**15**	**5**	**9**	**2**	**10**
Wilcoxon p	**0.002**	**<0.001**	**<0.001**	**<0.001**	**0.001**

*Significant results were shown in bold. t-tests and Wilcoxon tests were performed within the patient group. t-test t: the t value for the t-test result. t-test p: the p value for the t-test result. Same for the Wilcoxon test. Abbreviations are the same as in Table 3*.

The statistical tests were also repeated on clinical scores. Shapiro tests on sensory scores of the first and the second session rejected the null hypothesis. The intra-group paired *t*-tests, combined with the Wilcoxon test results, indicated that lower limb movement score and SCIM increased significantly (*P* < 0.05) after treatment (Table 1).

**Figure 3:**
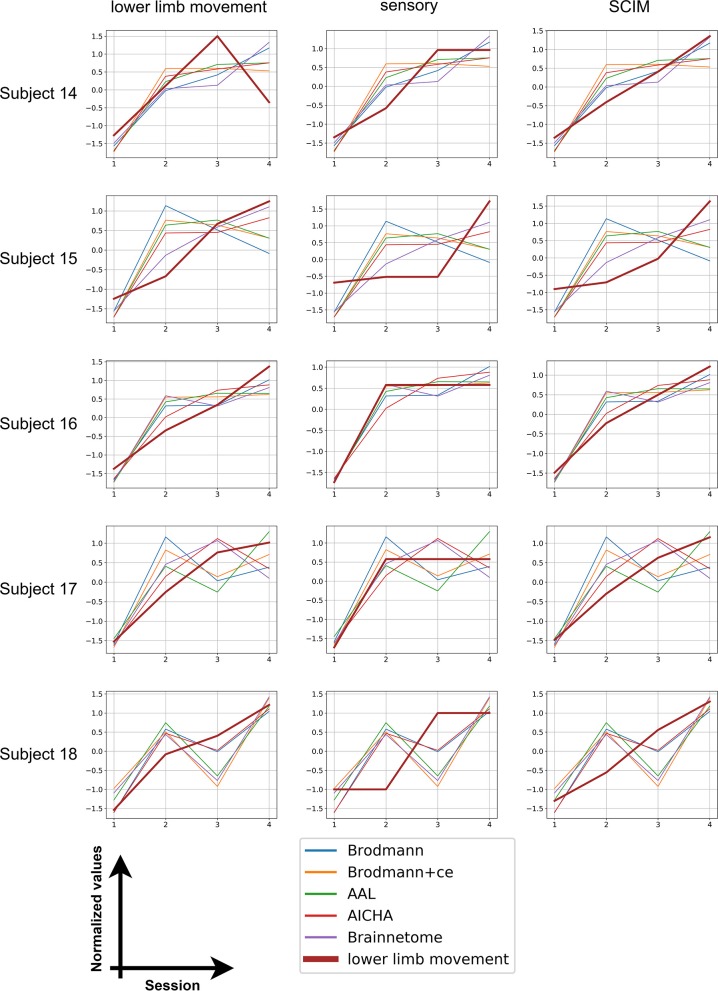
Longitudinal results. The bold brown line stands for clinical scores whereas thinner lines with five different colors represent the distance calculated from five different atlases.

We also calculated distance for the five subjects with four session data and plotted the distance with each clinical measurement. The results are shown in Figure 3. The score and distance values were normalized to have zero mean and unit variance and the distance values were reversed (that is, multiplied by −1) so that an increase in distance also means an increase in scores. It can be seen from the graph that all patients' scores show similar trend with distance, indicating the potential of using distance to predict rehabilitation outcomes.

## Discussion

We proposed a novel distance-based neurorehabilitation evaluation method and applied it to a dataset of SCI patients and HCs, utilizing five whole-brain atlases to validate our method. The testing results showed that the proposed method can reflect rehabilitation-induced functional changes. The changes found by distance in the multi-level brain networks built from five atlases are consistent with clinical measurements. Moreover, all of the rehabilitation outcome predictions for individual patients had a consistent trend with clinical scores. To our knowledge, this is the first time that rs-fMRI and linear SVM are applied to evaluate the rehabilitation progress and predict outcomes of SCI patients.

The prognostic value of resting-state FC was previously assessed by correlating FC with clinical scores and behavioral performance for groups of patients with stroke (44–47). Qin et al. (48) utilized support vector classifier and principal component analysis on resting-state FC to estimate the medication status of major depression patients after antidepressant treatment using a regression model. However, only a part of patients received second session MRI scanning and no longitudinal data (>2 sessions) were collected. Besides, few studies investigated outcome prediction of SCI patients after rehabilitation.

In our work, all patients received at least two sessions of MRI scanning and five patients were scanned two more times. The HCs, on the other hand, were only scanned once. In literature, measures of FC were suggested to vary during aging (49, 50) but were relative stable across time spans of 1–2 years (51). In our work, longitudinal scanning was mostly collected within 6 months and the FC was assumed to be stable. The healthy subject data were used to train a classifier and build up a healthy baseline, in order to measure how different the patients were from this baseline.

When building FC networks, we utilized brain atlas to define nodes, since it provides an objective node definition and is stable over different datasets. Unlike data-driven approaches, such as group independent component analysis (ICA), nodes defined by atlases do not change, which is preferable in rehabilitation evaluation. The node definition can not only alter the topological structure of the graph (network) but also influence the functional relationships between nodes. Some previous researches have noted the potential pitfalls of using atlases to define nodes in brain networks (34, 52). In addition, several studies found atlas-related differences in graph metrics of resting-state networks (53), ROI homogeneity (54), and structural networks (55). In this study, we obtained similar results in most experiments for all five atlases, indicating that our proposed method is not sensitive to the choice of node definition. However, we note that whether a certain atlas is suitable for whole-brain functional network classification or analyzing a specific disease is unclear. Future studies focusing on the choice of brain atlases could possibly facilitate the construction of multi-level FC networks.

During classifier training, we used LOOCV to estimate the accuracy and reported precision, sensitivity, and specificity along with accuracy. The parameter *C* in SVM training could affect the separating hyperplane. We repeated our experiments on a range of *C* values and the results were consistent. Also, the distance for each patient was calculated in a LOOCV framework. The use of LOOCV could alleviate the problem of overfitting and skewed dataset to some extent. The normality of distance values and scores was checked by the Shapiro test. Most tests did not reject the null hypothesis of normal distribution. Besides, we repeated the statistical analysis using non-parametric tests, which are supposed to work well for small sample sizes and not normally distributed data. Results obtained by the non-parametric tests corroborated with *t*-tests.

In order to evaluate the classifier performance, the second session data were used for testing. We observed a decreased accuracy for all atlases. Unlike a diagnosis problem where classification accuracy is critical, in our method, SVM is used to locate the separating hyperplane and calculate distance. As hypothesized, patients would become more similar to HCs after treatment, resulting in a shifted feature vector toward negative examples (HCs). This could be the cause of the decrease in accuracy. Besides, the training results of whole-brain features were not satisfying, indicating that feature selection is necessary when applying machine learning method to fMRI data.

We calculated distance of HCs at baseline and patient at two sessions. The distance of patients was found to decrease after treatment. According to our assumption, a decreased distance represents a closer position of feature vector to the negative samples, implying that the FC of the subject is becoming similar to HCs. The significant distance decline in the patient group indicated that the FC of the patient group recovered after treatment. On the other hand, the lower limb movement score and SCIM of patients also improved significantly, corroborating findings given by distance.

Unlike disease diagnosis, rehabilitation evaluation requires not only identifying the difference between groups of subjects but also reflecting the trend of multi-session data. The longitudinal data of five subjects were utilized to investigate the rehabilitation outcome prediction of our proposed method. All reversed distance has a consistent increasing trend as clinical scores from the first session to the fourth, indicating the improved function in patients after treatment. However, individual discrepancies exist. Among the five subjects, subject 16, whose last two scanning times were not far from treatment (4 and 6 weeks after inclusion), showed the most aligned variance in distance and scores. The scanning time gaps for subjects 14, 15, and 17 were similar, and fluctuations and inconsistencies were observed in the variation of distance. The scanning time of the last session of subject 18 was far away from the first session, which may influence the distance calculated from fMRI data. In fact, after 2 weeks' treatment, subjects returned home and stopped rehabilitation programs. Different self-exercising in these periods may be the cause of fluctuations in distance. Nevertheless, all subjects showed improved clinical scores in the fourth session compared with the first session, which can also be identified using distance. This proved that our method can reflect general trends of functional changes after rehabilitation treatment, but giving more precise prediction is still difficult. Future studies are needed to recruit more subjects as well as unify the follow-up revisit time gap.

There are several drawbacks in this study. Firstly, the number of samples and scanning sessions is limited. The correlation of longitudinal distance and scores only contained four sessions. As a result, no meaningful statistical correlation coefficient could be used to assess the consistency of distance and scores. It is also intriguing to investigate how the distance of longitudinal data of HCs would change in our method. At the time, we are recruiting more subjects to expand the dataset. More data collected during rehabilitation could further validate our findings.

Second, the accuracy might be over-optimized, since labels of samples were considered during feature selection. All data from the first scanning session were fed into the *t*-test filter, which may cause the classification algorithm to overfit the training dataset. Also, the significant threshold was set to 0.05 without multiple comparison correction. We chose to use a linear SVM instead of kernel SVM to alleviate the overfitting problem. Besides, we want to find a set of rich and consistent features that separates patients from HCs, instead of modifying the feature set whenever new data arrives. The original uncorrected threshold could reveal as many potential significant connections as possible. As reported in the Results section, the empirical discovery rate was higher than the significant level and were consistent across brain atlases for each disease. Yet, the uncorrected significant level indeed resulted in high dimensionality of the feature space, introducing redundant features, and causing possible pitfalls in the classifier training. On the other hand, the *t*-test filter is a very simple feature selection method that could be replaced by more sophisticated and robust method in the future.

Third, we used SCI patients to validate our method, but how it performs on patients with brain lesions remains unknown. The traumatic, ischemic, or hemorrhagic lesions due to traumatic brain injury, stroke, or other diseases could impact and alter FC. The lesion location and type should be considered when building brain networks and extracting features in such circumstances. In addition to the whole-brain network, a local network built at the lesion location and surrounding area could possibly capture disease-specific features.

## Conclusion

In this paper, we proposed a distance-based neurorehabilitation evaluation method, using rs-fMRI and linear SVM classifier, to investigate brain function changes following rehabilitation program. We proved that the distance results were consistent with clinical scores, indicating potential value in neurorehabilitation evaluation and outcome prediction. However, more robust feature extraction and selection techniques are needed before the method could be used in clinical practice. We hope that this paper would give rise to more innovations to tackle the problem of neurorehabilitation evaluation and outcome prediction.

## Data Availability Statement

The whole-brain functional connectivity matrices used in this study are available on request to the corresponding author.

## Ethics Statement

The studies involving human participants were reviewed and approved by the Ethics Committee of Beijing Tsinghua Changgung Hospital of China (IRB No. 2015-002). The patients/participants provided their written informed consent to participate in this study.

## Author Contributions

YG is responsible for conducting experiments and writing the manuscript. WD designed and conducted experiments. YP and QW recruited subjects and collected data.

### Conflict of Interest

The authors declare that the research was conducted in the absence of any commercial or financial relationships that could be construed as a potential conflict of interest.

## Footnotes

^1^http://circos.ca

## Appendix

For illustration purposes, we plotted the significant connections using Circos. The outer ring in the Circos plot represents brain regions and is separated into frontal, parietal, temporal, and occipital lobes, with or without cerebellum. Each tick in the ring stands for a brain region. The ticks are arranged so that frontal regions appear on the top of the graph. The lines inside Circos plots represent connections between two regions.

In our study, during *t*-test filtering, each connection was tested for significant difference between all patients and HCs at baseline. Connections were sorted by absolute *t* value and the top 100 connections were plotted in the graph. A red line means that for this connection, patients were significantly higher than HCs and a blue line means the opposite. The same procedure was repeated for all five atlases.
